# Case report: Application of three-dimensional technologies for surgical treatment of portosystemic shunt with segmental caudal vena cava aplasia in two dogs

**DOI:** 10.3389/fvets.2022.973541

**Published:** 2022-08-12

**Authors:** Jinsu Kang, Myungryul Yang, Yonghwan Kwon, Chorok Jeong, Namsoo Kim, Suyoung Heo

**Affiliations:** ^1^Department of Surgery, College of Veterinary Medicine, Jeonbuk National University, Iksan-si, South Korea; ^2^Department of Internal Medicine, College of Veterinary Medicine, Jeonbuk National University, Iksan-si, South Korea

**Keywords:** caudal vena cava aplasia, portosystemic shunts, three-dimensional, dog, vascular model

## Abstract

This case report describes the application of three-dimensional (3D) technologies for the surgical treatment of portosystemic shunt (PSS) with segmental caudal vena cava (CVC) aplasia. Two client-owned dogs were diagnosed with PSS along with segmental CVC aplasia using computed tomography. Through 3D volume and surface rendering, the vascular anatomic anomaly of each patient was identified in detail. A patient-specific 3D vascular model was used for preoperative planning. According to the plan established based on the 3D rendered image and printed model, shunt occlusion was performed using cellophane banding in the first case. An ameroid constrictor was used in the second case. Both patients showed good recovery without any clinical symptoms or complications. The use of 3D technologies in small animals has many advantages, and its use in vascular surgery, as in these cases, is also a therapeutic option worth considering.

## Introduction

Segmental caudal vena cava (CVC) aplasia is a rare congenital vascular anomaly in dogs (1, 2). This anomaly is also called azygos continuation of the CVC, CVC uniting with the azygos vein, and CVC absence, interruption, or discontinuation (1, 2). In this anomaly, the CVC segment between the kidneys and liver does not form. The renal and postrenal caval blood is instead shunted toward an enlarged azygos vein, ensuring functional blood return to the heart (3, 4). It is usually confirmed as an incidental finding during imaging tests to diagnose portosystemic shunts (PSS) or anatomical dissection during laparotomy (4, 5). Segmental CVC aplasia combined with PSS has not been reported frequently in veterinary medicine (2, 4, 6, 7).

Advanced imaging techniques, such as computed tomography (CT) and magnetic resonance imaging, have become the gold standard when high-resolution or three-dimensional (3D) images are indicated in establishing a diagnosis and planning surgery (8–10); 3D reconstruction and printing of cross-sectional images have been advancing for decades. They provide more precise views of anatomical structures that are convenient for accurate surgical planning (11–13). The use of 3D technology is also increasing in the field of vascular surgery in human and veterinary medicine (14–17).

Surgical treatment of porto-azygos shunt with segmental CVC aplasia has not been reported in many cases in veterinary medicine owing to poor prognosis (18). This case report describes the successful surgical treatment of PSS with segmental CVC aplasia aided by 3D technology in two dogs.

## Case description

### Case no. 1

A 1-year-old castrated male poodle (4.3 kg) presented to the Animal Medical Center of Jeonbuk National University with a history of seizure and ptyalism. Physical examination results were normal, and complete blood count (CBC) results were within normal ranges. Serum chemistry abnormalities included low concentration of creatinine (0.21 mg/dL; reference range (RR), 0.4–1.4 mg/dL), urea nitrogen (8.5 mg/dL; RR, 9.2–29.2 mg/dL), and total protein (4.2 g/dL; RR, 5–7.2 g/dL) and high concentration of alanine aminotransferase (149 U/L; RR, 17–78 U/L) and alkaline phosphatase (532 U/L; RR, 47–254 U/L), and hyperammonemia (157 μmol/L; RR, 0–99 μmol/L).

**Figure 1:**
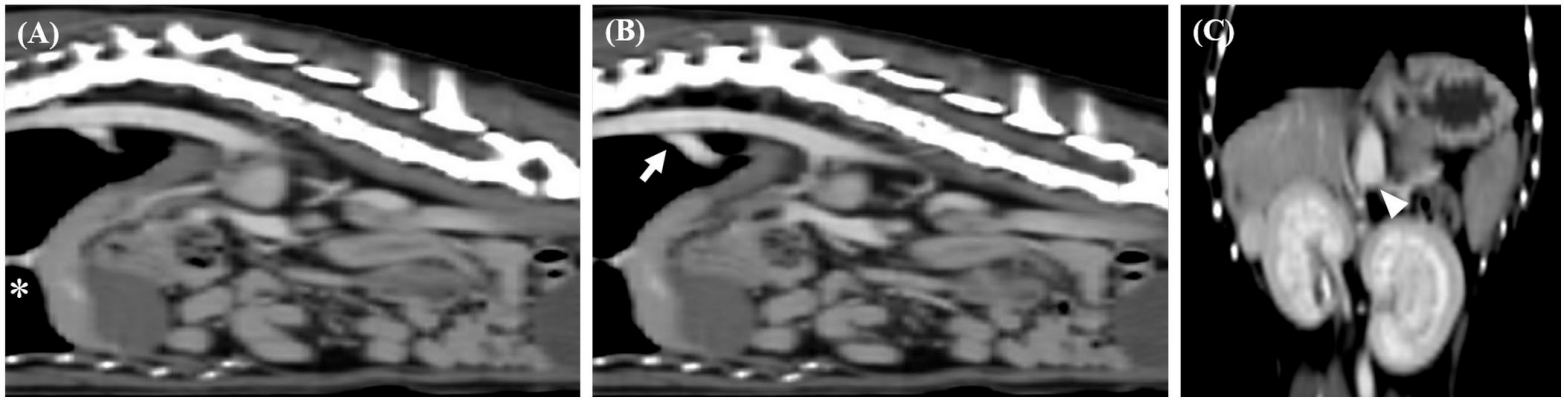
Sagittal computed tomography images from the late post-contrast series of cases 1. **(A,B)** Segmental caudal vena cava aplasia (asterisk) and portosystemic shunt (arrow) were observed. Dorsal computed tomography images from the late post-contrast series of cases 1. **(C)** The white arrow head indicates caudal portion of the shunt.

Radiographic examination (HF-525 PLUS; Ecoray, Seoul, Korea) revealed an enlarged right azygos vein and microhepatica. CT angiography was performed using a 16-row multi-detector CT scanner (Alexion, TSX-034A, Canon Medical Systems Europe B.V., Zoetermeer, Netherlands) with the patient anesthetized to diagnose and evaluate PSS. CT revealed segmental CVC aplasia (right lateral cavo-right-azygos shunt) and a porto-azygos shunt (Figure 1).

For seizure control, preoperative medications, including levetiracetam (20 mg/kg orally every 8 h for 2 weeks) and lactulose (1 mL/kg orally every 12 h for 2 weeks), were administered. For hepatoprotective therapy, medications, including ursodeoxycholic acid (10 mg/kg orally every 12 h for 2 weeks), S-adenosylmethionine (20 mg/kg orally every 12 h for 2 weeks), and silymarin (15 mg/kg orally every 12 h for 2 weeks), were administered. After confirming the return of serum ammonia level within a normal range, surgery was planned with the consent of the owner.

#### 3D modeling and printing

**Figure 2:**
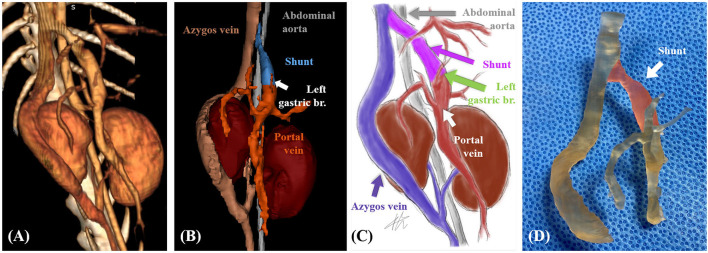
Case 1. Volume-rendered three-dimensional (3D) projection of the computed tomography (CT) angiography study **(A)**. Surface-rendered 3D projection of the CT angiography study **(B)**. Illustrations of vascular anomaly **(C)**. 3D printed vascular model (areas marked in transparent red color indicate shunt) **(D)**.

Mimics (version 23.0; Materialize NV, Leuven, Belgium, 2020) was used to accurately identify the abdominal vascular structure of the patient through volume and surface rendering. The left gastric branch of portal vein could be identified more clearly through surface rendering, and the blood vessels were separated to facilitate 3D printing (Figures 2A,B). Based on 3D rendering, the structure was clearly identified by illustration of blood vessels and surrounding organs (Figure 2C).

Patient-specific 3D vascular models (Figure 2D) were printed using a resin 3D printer (Pixel One, Zerone, Gyeonggi, Korea), and dental surgical guide resin (SG-100, Graphy, Seoul, Korea) was used. After printing, the models were washed and dried for 30 min and cured using UV light at a wavelength of 405 nm for 60 min (3DP-100S, CUBICON, Gyeonggi, Korea).

#### Surgical procedure

Rehearsal surgery was performed using a 3D vascular model before surgery. As the shunt passed through the diaphragm, we planned to approach the diaphragm through the abdominal cavity. In order to minimize the influence of surrounding blood vessels, including the gastric branch, the occlusion location closest to the diaphragm was determined.

**Figure 3:**
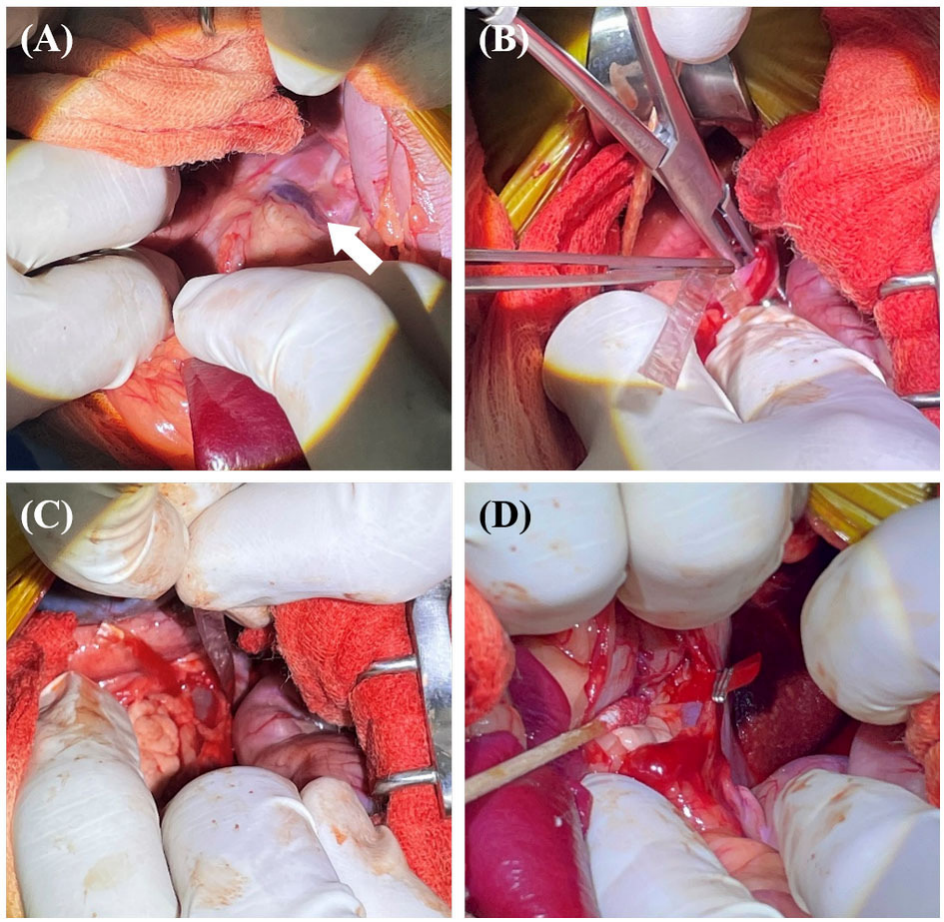
The surgical procedure for case 1. Approach the diaphragm to secure visibility around the shunt (white arrow) **(A)**. The band of cellophane was placed around the shunt with minimal dissection **(B,C)**. The band was placed in contact with the wall of the shunt without decreasing the diameter of the shunt. Vascular clips were used to secure the band in place **(D)**.

After following a standard premedication protocol and anesthesia induction, a midline laparotomy was performed. While viewing the sterilized 3D vascular model during surgery, the shape of the blood vessel and planned occlusion location were reconfirmed. A porto-azygos shunt from the right azygos vein of the thoracic cavity to the abdominal cavity was found near the diaphragm, and cellophane banding (3 mm wide triple-layer bands) was performed with minimal dissection around the shunt (Figure 3).

#### Follow-up

**Figure 4:**
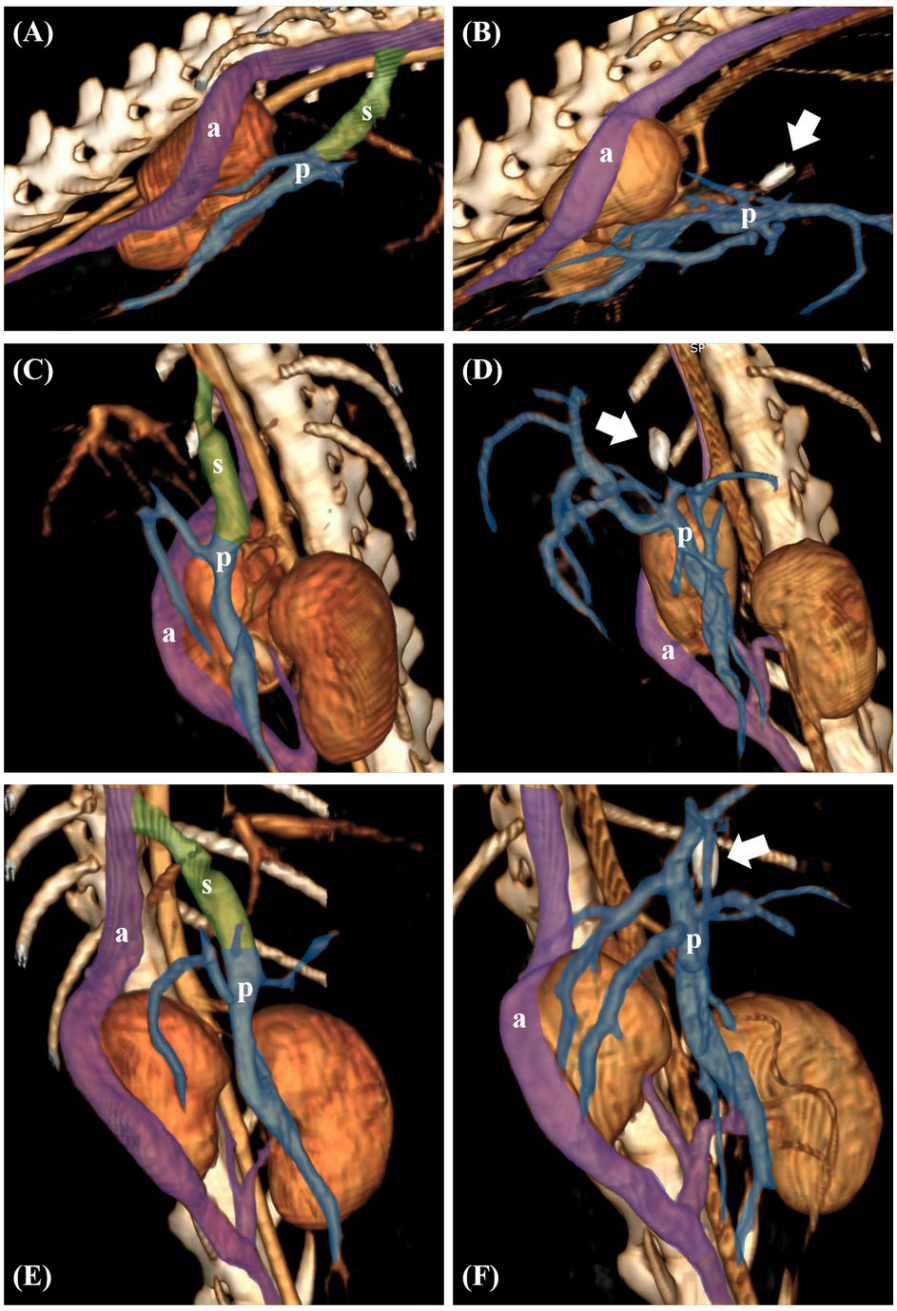
Preoperative volume-rendered three-dimensional (3D) images in case 1 **(A,C,E)**. Postoperative volume-rendered 3D images in case 1 revealed complete occlusion of the shunt (white arrow indicates surgical clip) **(B,D,F)**. The transparent green area represents the shunt (s). The transparent purple area represents the right azygos vein (a) and the transparent blue area represents the portal vein (p), respectively.

The patient received post-operative medications, including antibiotics (amoxicillin and clavulanic acid, 12.5 mg/kg orally every 12 h for 1 week), a prescription diet (low-protein diet for 2 months), and lactulose (1 mL/kg orally every 12 h for 2 months). There were no specific clinical symptoms, such as seizures post-surgery, and a CT scan 8 months after surgery revealed a completely occluded shunt (Figure 4).

### Case no. 2

**Figure 5:**
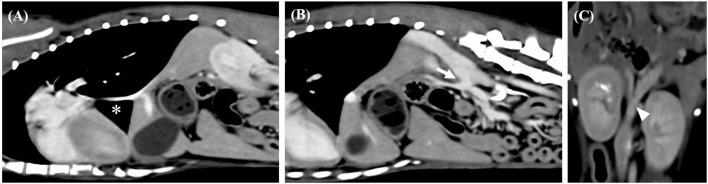
Sagittal computed tomography images from the late post-contrast series of cases 2. **(A,B)** Segmental caudal vena cava aplasia (asterisk) and portosystemic shunt (arrow) were observed. Dorsal computed tomography images from the late post-contrast series of cases 2. **(C)** The white arrow head indicates caudal portion of the shunt.

An 11-month-old intact female Mongrel (5.2 kg) was presented to the Animal Medical Center of Jeonbuk National University for screening. Physical examination results were normal, and the CBC results were within normal ranges. Serum chemistry abnormalities included low total protein concentration (3.9 g/dL; RR, 5–7.2 g/dL), high alanine aminotransferase (177 U/L; RR, 17–78 U/L) and alkaline phosphatase (521 U/L; RR, 47–254 U/L), and hyperammonemia (142 μmol/L; RR, 0–99 μmol/L). Radiographic examination revealed microhepatica. PSS was suspected based on the owner's choice, and a CT scan was performed. CT revealed segmental CVC aplasia (dorsal cavo-right-azygos shunt) and a porto-azygos shunt (Figure 5).

The patient received preoperative medications, including ursodeoxycholic acid (10 mg/kg orally every 12 h for 2 weeks), S-adenosylmethionine (20 mg/kg orally every 12 h for 2 weeks), silymarin (15 mg/kg orally every 12 h for 2 weeks), and lactulose (1 mL/kg orally every 12 h for 2 weeks). After confirming the return of serum ammonia level within a normal range, surgery was planned with the consent of the owner.

#### 3D modeling and printing

**Figure 6:**
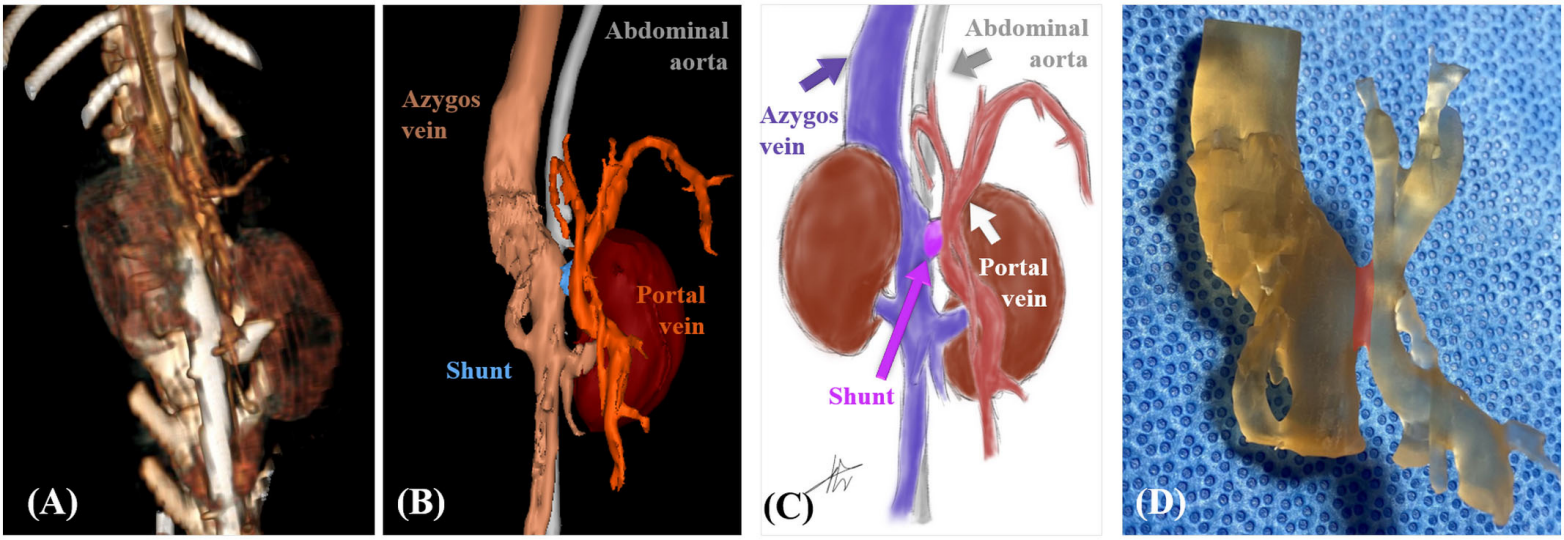
Case 2. Volume-rendered three-dimensional (3D) projection of the computed tomography (CT) angiography study **(A)**. Surface-rendered 3D projection of the CT angiography study **(B)**. Illustrations of vascular anomaly **(C)**. 3D printed vascular model (areas marked in transparent red color indicate shunt) **(D)**.

Mimics was used for accurate identification of the patient's abdominal vascular structure through volume and surface rendering. The 3D modeling provided visual confirmation of the absence of abnormal blood flow to the abdominal organs, except for the shunt (Figures 6A,B). Based on 3D rendering, the structure was clearly identified by illustration of blood vessels and surrounding organs (Figure 6C).

Patient-specific 3D vascular models (Figure 6D) were printed using a resin 3D printer, and a dental surgical guide resin was used. The materials and methods used were the same as those in Case 1.

#### Surgical procedure

Rehearsal surgery was performed using a 3D vascular model before surgery. As the shunt was located between the kidneys, an incision was made from the umbilicus to the pubis.

**Figure 7:**
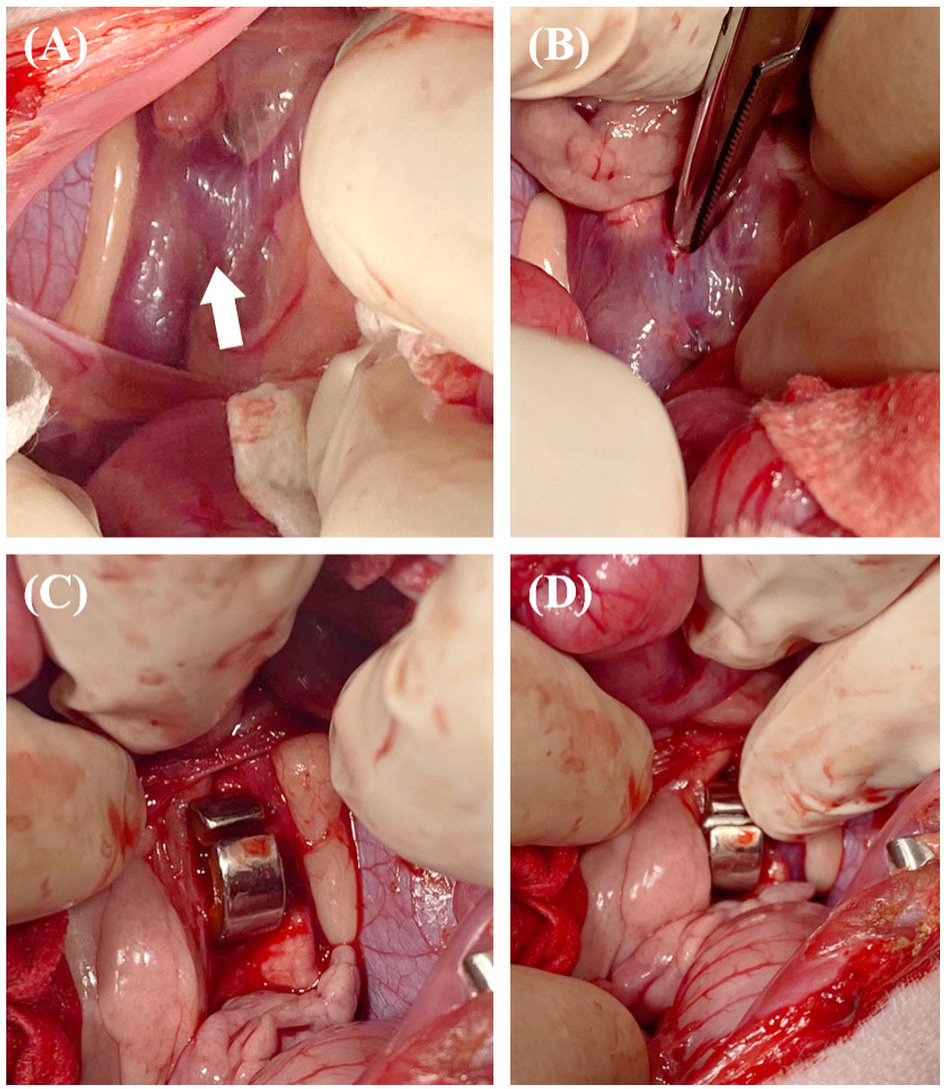
The surgical procedure for case 2. Approach between the kidneys to secure visibility around the shunt (white arrow) **(A)**. The dissection performed was sufficient to place an ameroid constrictor **(B)**. The constrictor was placed around the shunt **(C,D)**.

After following a standard premedication protocol and anesthesia induction, a midline laparotomy was performed. While viewing the sterilized 3D vascular model during surgery, the shape of the blood vessel and planned occlusion location were reconfirmed. A porto-azygos shunt was found between the kidneys, and an ameroid constrictor (5 mm internal diameter ring) was placed with dissection around the shunt (Figure 7).

#### Follow-up

The patient received post-operative medications, including antibiotics (amoxicillin and clavulanic acid, 12.5 mg/kg orally every 12 h for 1 week), a prescription diet (low-protein diet for 2 months), and lactulose (1 mL/kg orally every 12 h for 1 month). No specific clinical symptoms were observed post-operatively.

## Discussion

These cases report the application of 3D technologies for the surgical treatment of PSS with segmental CVC aplasia in two dogs. Segmental CVC aplasia can be divided into seven types (2). In the previous studies, PSS concurred in 10 of 37 dogs with segmental CVC aplasia (2–4, 6, 19–21). Most cases of PSS concurred with segmental CVC aplasia are prone to drainage into the azygos vein (6). According to previous studies, porto-azygos shunt with CVC aplasia is atypical (22). Therefore, it might not be easily distinguished during surgery because of the atypical and complex abdominal vascular anatomy (23). As incorrect surgical ligation might be fatal, a more accurate assessment of the abdominal vascular structure should be performed in patients with such atypical vascular anomalies (23, 24).

In these cases, segmental CVC aplasia was diagnosed incidentally through a CT scan as previously reported (2, 4). Both patients had clinical symptoms of PSS, and there were no clinical symptoms related to segmental CVC aplasia. Accordingly, surgery was decided upon with the consent of the owners. Volume and surface rendering in 3D imaging were additionally performed to accurately identify vascular anomalies in the abdominal cavity and to perform preoperative planning. The microvascular anatomy of the lesion was confirmed using surface rendering.

In case 1, 3D reconstruction images confirmed that the blood flow in the surrounding organs was not disturbed owing to shunt occlusion. Through 3D surface rendering, the presence of a left gastric branch of portal vein was confirmed in the area close to the planned occlusion location. If an ameroid constrictor is used, the possibility of constrictor movement or shunt kinking is considered because the structure of the shunt descends from the dorsal to the ventral direction. It was determined that this could affect the blood flow of other blood vessels, as well as the left gastric branch of portal vein; therefore, minimal dissection around the shunt was done along with the use of cellophane banding as an occlusion material. The 3D reconstruction images confirmed that blood flow in the surrounding organs was not disturbed by shunt occlusion in case 2 as well. The shunt was located between the portal vein and the right azygos vein and there were no other portal vein branches that could be affected by the occlusion like case 1. Also, due to the short length of the shunt, it was assumed that the possibility of constrictor movement was low, so an ameroid constrictor was determined as the occlusion material.

In addition, a patient-specific 3D vascular model was printed for a better understanding of the anatomy of the lesions and to check the feasibility of surgery in these cases. Conventional reconstructed 3D digital images only provide a rough approximation of the underlying anatomy and are considered insufficient, especially in complex vascular pathologies (25). In 2001, 3D prototyping was first applied in the field of vascular surgery and has since been successfully applied (14). Although the application was initially limited to large vessels (thoracic and abdominal aorta), there have been an increasing number of publications over the past decade involving small and medium vasculature such as the splenic and internal mammary artery (26, 27). Printed patient-specific 3D vascular models provide outstanding anatomical insights for simulating surgical procedures (28), which are advantageous in terms of patient safety, with reduced anesthesia and operation time (29). It also shortens recovery time, reduces blood loss during surgery, and consequently results in a lesser cost of health services (28, 30).

Although their application is limited to small animal orthopedics in veterinary medicine, the proposed benefits of 3D printing for surgical planning of complex cardiovascular diseases in humans may be related to the management of small animals with vascular malformations, such as intrahepatic portal-systemic shunts (17). 3D printing was used to visualize abnormal coronary arteries in six dogs with pulmonary stenosis and in surgical planning to correct concurrent patent ductus arteriosus, aberrant left subclavian artery, and right aortic arch in one dog (31, 32). In a previous report, a 3D print derived from preoperative CT angiography was used to create a checklist before the operation, select clamp and staple devices, and plan the surgery (31).

Similarly, in these cases, rehearsal surgery using a 3D patient-specific vascular model, the incision site of the laparotomy for accessing the shunt, and the approach direction of surgical instruments considering the structures around the shunt were planned before surgery. In addition, as the reconstructed blood vessels were printed more accurately based on surface rendering, the location of occlusion and the occlusion material that could minimize the effect of shunt occlusion on other normal blood vessels could be determined. In the preoperative consultation with the owners, the vascular model was used to explain the abnormalities of vascular structure, surgical procedure, and post-operative complications. They showed better understanding and satisfaction with consultation using 3D printed models rather than that done using conventional CT and 3D rendering images.

There were some limitations to using the 3D vascular model in this case report. First, the number of cases was limited. Further prospective comparative studies are needed to evaluate the benefits of 3D patient-specific vascular model. Second, additional time is required to produce the 3D vascular model, and the cost must be considered. Finally, printing, post-processing, curing and sterilization, can also modify the 3D vascular model (33). Therefore, an error may occur in measuring the exact diameter of a blood vessel through the 3D vascular model.

This case report describes the successful surgical treatment of PSS with segmental CVC aplasia in two dogs using 3D technologies. We were able to identify the exact vascular anatomy of the patients and optimize preoperative planning by using 3D technologies. Although 3D technologies have been predominantly used in hard-tissue surgery, these cases suggest the possibility of using 3D technologies in the field of vascular surgery in veterinary medicine.

## Data availability statement

The original contributions presented in the study are included in the article/supplementary material, further inquiries can be directed to the corresponding author.

## Ethics statement

Ethical review and approval was not required for the animal study because our manuscript is based on clinical cases. Written informed consent was obtained from the owners for the participation of their animals in this study.

## Author contributions

JK and SH: conception, design, and draft of the manuscript. JK, YK, and SH: main clinician during case presentation and performed surgery. CJ: internal medicine consultation. JK, MY, NK, and SH: analyzed and interpreted the data. JK, NK, and SH: revised the article for intellectual content and final approval of the completed article. All authors have contributed to the manuscript and approved the submitted version.

## Funding

This research was financially supported by the Ministry of Small and Medium-Sized Enterprises (SMEs) and Startups (MSS), Korea, under the Regional Specialized Industry Development Plus Program (R&D, S3244754) supervised by the Korea Technology and Information Promotion Agency (TIPA). This paper was supported by Jeonbuk National University Animal Medical Center.

## Conflict of interest

The authors declare that the research was conducted in the absence of any commercial or financial relationships that could be construed as a potential conflict of interest.

## Publisher's note

All claims expressed in this article are solely those of the authors and do not necessarily represent those of their affiliated organizations, or those of the publisher, the editors and the reviewers. Any product that may be evaluated in this article, or claim that may be made by its manufacturer, is not guaranteed or endorsed by the publisher.

## Abbreviations

CVC: caudal vena cava
CT: computed tomography
3D: 3-dimensional
PSS: portosystemic shunt
CBC: complete blood count
RR: reference range.
